# Measuring private transfers between generations and gender: an application of national transfer accounts for Austria 2015

**DOI:** 10.1007/s10663-022-09542-z

**Published:** 2022-05-13

**Authors:** Bernhard Hammer, Alexia Prskawetz

**Affiliations:** 1 TU Wien/Institute of Statistics and Mathematical Methods in Economics, Vienna, Austria; 2 Wittgenstein Centre (Univ. Vienna, IIASA, VID/ÖAW), Vienna, Austria

**Keywords:** Generational economy, National transfer accounts, Intra-family transfers, D13, J16

## Abstract

Few data sources provide information on private transfers between generations and gender. We use a novel approach based on the National Transfer Accounts methodology to estimate the value of intra-family transfers between generations by age, gender and parental status in Austria 2015. The paper considers monetary transfers together with transfers of consumption goods and transfers of services produced by non-market work. Our results show that parents use one third of their disposable income and up to four hours of daily non-market work for their children. The total size of the intra-family transfers corresponds to 38 per cent of primary income.

## Introduction

Families provide for the needs of children and offer social protection for their adult members. However only few data sources include information on the extent and the direction of intra-family redistribution. To close this data gap for Austria, we apply the National Transfer Accounts methodology and estimate total transfers of income and of unpaid services within families as well as mean values by age, gender and by parental status. This information allows for a better assessment of the economic situation of parents and the economic challenges that families are facing.

Characteristic for the life course in modern societies are long periods of economic dependency in childhood and old age, when consumption exceeds own production. Three channels for financing the difference between consumption and production can be distinguished: private transfers, public transfers and asset-based reallocation of resources.^1^ In European welfare states, public transfers are mainly directed at the elderly population. For children the most important type of support are private transfers from parents and other family members. Because private transfers take place almost exclusively between members of the same family, we use the terminology private transfers and intra-family transfers interchangeably.

While public transfers are regularly measured and analysed by public organizations and statistical institutes (e.g. OECD 2019; Commission 2021), few data sources and studies focus on transfers within families. The costs of children that are covered by their parents were only recently estimated in Austria (Bauer et al. 2021), for the first time since 2003 (Guger et al. 2003). This new study however does not collect data directly, as suggested by Neuwirth and Halbauer (2018), but applied indirect methods based on the consumer expenditure survey (CES). According to the study, estimated costs of children range between 300 Euros per month up to 1500 Euros, depending on the household structure and age of the children.

National Transfer Accounts (NTAs) and National Time Transfer Accounts (NTTAs) provide comprehensive information about generational aspects of the economy, including age and gender-specific information on income, public and private transfers, consumption and saving (Lee and Mason 2011). Private transfers in NTAs consist mainly of monetary transfers and transfers of consumption goods between household members, with parents financing the consumption of children as most important component. For these transfers we use the term *market transfers*, because by generating income and purchasing goods and services these transfers involve market transactions. NTTAs integrate non-market production, transfers and consumption into the NTA system (Donehower 2019). The transfers captured in NTTAs are referred to as *non-market transfers*. Non-market transfers consist of services that are produced for members of the own household or provided free of charge to other households. The most important components of these non-market transfers are childcare and household services such as cooking and cleaning.

Research based on NTAs and NTTAs provide novel insights on the role of families in the intergenerational transfer system. In all countries around the world consumption of children is financed mainly by private market transfers (NTA 2016) and in some Asian countries they also play a significant role in old age provision (e.g. Ladusingh and Maharana 2018). While most NTA-based research focuses on age-averages, recent contributions analyse differences by gender and socio-economic status that explicitly show the role of families for intergenerational redistribution. Abio et al. (2021) disaggregate NTAs by parental status for four European countries. Their results show that for parents the net contributions to intra-family transfers are of about the same size as their net contributions to the public transfer system. Note that we use the term *contributions* for transfers paid and *benefits* for transfers received. All age groups contribute to transfers and receive transfers; with *net contributions* we refer to contributions less benefits and, likewise, with *net benefits* we refer to benefits less contributions.

Additional to the transfers of income and consumption goods, parents use a large amount of time to provide services to their children. In their first two years of life children require between 5 and 7 hours of non-market work per day (Vargha et al. 2017; Zagheni et al. 2014). By accounting for market- and non-market work, Gál et al. (2018) find that the value of public and private transfers to children is larger than the value of transfers to elderly persons. Hammer et al. (2018) show that private market and non-market transfers to children are a central component of the *generational contract*, referring to the economic flows that ensure the sustainability of intergenerational redistribution in the long run. The size of the private and public transfers to the child generation determines their potential to finance public transfers to the elderly population once they themselves enter the labour market. The results in Hammer et al. (2018) indicate a current imbalance between investments in children and generous transfers to the elderly population in 16 European countries.

One important focus of NTA and NTTA research are gender differences, because the information on private market and non-market transfers together with public transfers illustrate the gendered organization of intergenerational redistribution. Basic gender patterns are found to be similar in all countries: men provide more public transfers and private market transfers to children, women provide most of the non-market transfers within families (Hammer et al. 2020). For women, non-market work peaks at childbearing age and in retirement with values of more than 5 hours of non-market work per day. However, the specific role of men and women in the intergenerational transfer system differs widely across countries. In Spain, Italy and Slovenia, women use considerably more time for work activities than men and contribute more to the intergenerational transfer system (Hammer et al. 2020; Rentería et al. 2016). Austria is characterised by a gender-equal distribution of total working time (Zannella et al. 2019). However, also in Austria women carry out a much larger part of the non-market work. High part time rates among women in Austria and the lower amount of paid work results in lower income, lower contributions to the pensions system and consequently much lower yearly pensions for women (Eurostat 2020b). Because of their lower income, Austrian women receive positive net market-transfer from their partner at all adult live stages. By contrast, men receive positive non-market transfers from their female partner in later working life and retirement (Hammer et al. 2020).

The Austrian NTAs for 2015 provide novel and detailed information on private transfer contributions and benefits, including estimates of their total extent as well as estimates of mean values by age, gender and parental status. Before 2015, NTAs for Austria have been compiled for 1995, 2000, 2005 and 2010 (Hammer 2014). Furthermore, Austria is included in the European NTA and NTTA dataset that has been generated within the AGENTA research project for the year 2010 (Istenič et al. 2016; Vargha et al. 2017).^2^ Abio et al. (2021) provide NTA results by education level and for parents and non-parents in four European countries. These former NTA data provide age- and gender-specific information on private net transfers only, i.e., they do not distinguish between contributions and benefits, and they estimate either market or non-market transfers, but do not integrate them into a single measure. The Austrian NTAs for 2015 use a novel approach to combine income data and consumption data, which allows to distinguish between contributions and benefits for intra-family transfers. Furthermore, the approach enables a breakdown of intra-family transfers not only by parental status, but also disaggregated by age of the youngest child. Based on the Austrian NTAs for 2015 we address the following questions: How large are intra-family transfers and how do they redistribute across age and gender? Taking into account the net contributions to private market transfers, how is the economic situation of parents compared to childless adults? As we illustrate, Austrian NTAs for 2015 enable a profound analysis of the role of the family in the intergenerational transfer system.

Understanding the role of families in the generational economy enhances our understanding of current economic and demographic developments. The financial crisis and sovereign debt crisis affected the young population and families much stronger as compared to older generations. Income of the younger population stagnated or even declined during the last decade, while it increased considerably for the older population (Rocha-Akis et al. 2019). This development resulted in a considerable decline of poverty rates among the population 60+, while poverty rates remained at comparably high levels for younger households, especially families with children (Eurostat 2020a). The economic crisis due to COVID-19, population ageing and the retirement of the baby-boomer will additionally increase the economic pressure on young generations in the coming years. Knowledge about the role of private transfers in the generational economy will improve our understanding of economic and social developments. These include a better understanding of the patterns of poverty and the individuals, households and families that are most vulnerable to poverty risk. Such understanding is a prerequisite for reforming welfare systems in a way that promotes sustainability and generational equity.

## Methodology and data

This chapter provides an overview of the general NTA and NTTA methodology and describes in detail the estimation of private transfers within households for Austria in 2015. The basic NTA methodology is described in detail in the manual of the UN (UN, 2013) and the NTTA methodology in Donehower (2019). Hammer (2020a) provides the description of data and specific methods for estimating NTAs for Austria 2015 except the details for private transfers.

### National transfer accounts: a general overview

NTA data provide information on age-specific means of income, public transfers, private transfers, consumption and saving. The broad estimation strategy for NTAs is, first, to derive the aggregate values from National Accounts data and related sources. Aggregate values refer to the quantities for the total economy, i.e. total labour income or total consumption in Austria 2015. In the second step the distribution over age groups and genders is estimated using survey- and administrative data. All age profiles are adjusted by an appropriate factor, to ensure that the per-capita averages for each age group summed up over the total population match the aggregate values. Likewise, gender-specific averages are adjusted so that the sum of male and female-specific values in each age group equals the age group total. In a third step transfers within families are estimated. These estimates of intra-family transfers are based on the difference between consumption and income.

Table 1 illustrates the central patterns of NTAs for Austria 2015. The average yearly primary income is highest in the age group 25-59 with more than 45,000 Euros on average. Primary income refers to income generated by direct participation in the production process and asset income. On average, a 25-59-year-old pays more than 13,000 Euros to children and elderly persons via net public transfers, and about 4,000 Euros to children in form of net private transfers. For children and young adults aged 0-24 the private transfers are the most important source of income. They receive more than 8,000 Euros of net private transfers per year, and more than 6,000 Euros of net public transfers, with education as main component. Public pensions and health services are the largest components of public transfers and directed mainly to the retired population. In total, the population 60+ receive more than 17,000 Euros of net public transfers, on average. Adjusted disposable income (including public transfers in kind) is increasing with age. While adjusted disposable income of the population below 25 is less than 20,000 Euros, it is more than 34,000 Euros for the population 60+. Consequently, the population 60+ has the highest level of total consumption (public + private) and the highest level of saving.

**Table 1:** National transfer accounts for Austria 2015: average yearly values in Euro for three age groups

	Age 0–24	Age 25–59	Age 60+
**Primary income (labour + asset)**	**5289**	**45,449**	**17,185**
Net public transfers benefits	6315	− 13,746	17,682
Net private transfers benefits	8301	− 3986	− 573
**Adjusted final disposable income**	**19,906**	**27,717**	**34,294**
Private consumption	10,265	18,554	20,484
Public consumption	10,100	5517	8956
Residual (saving)	− 459	3645	4853
Population	2,236,257	4,321,608	2,126,450

Notes: Net transfers benefits are defined as transfers received less transfers paid

Age and gender-specific NTA data for Austria is publicly available through the Austrian Social Science Data Archive (Hammer 2020b) and through the data base of the NTA project at www.ntaccounts.org. We changed the methodology for estimating private transfers in the course of writing and revising this paper, resulting in small differences between the data presented in this analysis and the NTA data included in the databases. The methodological changes are related to the imputation of consumption in income data, the original methodology is described in Hammer and Prskawetz (2020).

### Microdata for the estimation of private transfers

Intra-family transfers in NTAs are not measured directly, but are based on the difference between income and consumption of individuals. It is assumed that consumption is financed through transfers from other household members, if own income is not sufficient. These transfers consist mostly of flows from parents to children and, in gender-specific NTAs, of transfers between partners with large differences in income. NTAs focus on ”current transfers”, i.e. regular transfers. Wealth transfers, such as bequests or dowries, are therefore not included. Although wealth transfers play an important role in intergenerational redistribution, the limited data availability prevented so far the extension of NTAs with wealth and wealth transfers.

The estimation of intra-family transfers in NTAs relies on micro-data that represents the household structure and contains information on the consumption of households and the income of individual household members. However, in most countries, microdata that include both, information on consumption and the income of individual household members, are not available. When income and consumption are from different datasets, the NTA manual (UN, 2013) suggests to impute age-specific means of the missing income and/or missing consumption into microdata that represents the household structure. Estimates of intra-family transfers are then based on these imputed data. Such an approach has been chosen for Austrian NTAs for the years 1995, 2000, 2005 and 2010, which impute age-specific means of income (from tax statistic) and consumption (from the consumer expenditure survey) into data from the micro-census.

The NTA manual is clear about the weaknesses of NTAs that are based on microdata with imputed means, among them a bias in the estimates of transfers paid and transfers received and the impossibility to use these data to analyse subtypes of households. For example, many families with young children are characterized by large transfers of income from fathers to mothers, as mothers specialize in unpaid non-market work and fathers in market work. Age-specific averages of income greatly overstate the income of mothers and therefore underestimates the extent of intra-family transfers. Furthermore, to calculate age-profiles for sub-types of households, the differences across subgroups must be reflected in the imputed values. Abio et al. (2021) impute age-averages of consumption by subgroups of households, distinguishing by age, gender, education and family type. The disadvantage of this approach is that these estimates are partly based on few observations and require broad categories. For example, Abio et al. (2021) only distinguish between families with dependent children and without dependent children, ignoring the large differences within these groups, for example by age of the children.

The estimates of intra-family transfers and the scope of the NTA analysis can be greatly improved by combining data on income and consumption in a way that maintains the relation between household characteristics, income, and consumption. In particular, the imputation needs to account for those characteristics that have the greatest impact on consumption, most notably income. Such an approach enables the separate estimation of transfers paid and received and the analysis of intra-family transfers by characteristics other than age and gender. Since microdata with individual income is in most countries available the problem reduces to the imputation of consumption into income data.

### Imputing consumption into income data

A range of different methods have been developed for imputing information on consumption into income data. In particular, we can draw on the work for integrating indirect taxes (consumption taxes) into the European tax-benefit simulation model EUROMOD, which is based on EU-SILC. For the 2015 NTAs, we use the information on consumption from the Austrian consumer expenditure survey (CES)^3^ and impute this information into EU-SILC. The Austrian CES contains information on consumption of households as well as information on characteristics of households, such as household income, household size and the age of household members. EU-SILC contains information on income of each household member as well as characteristics of households. The information about households that is available in both surveys is used to estimate and impute consumption into EU-SILC data. Two broad types of imputation methods can be distinguished, regression based methods and matching methods. Regression models are estimated using CES, the predicted values of the consumption expenditure for specific household characteristics are then imputed into income data. The matching methods impute consumption expenditure into income data by using the values from CES-households with similar characteristics.

Decoster et al. (2007) compares different imputation methods, including both model-based and matching methods. In their analysis the model based methods produce a better fit of the imputed data to the observed data, while the matching methods are better in reproducing the original distribution of consumption. However, the differences between the distribution of consumption in the model-based imputations and the original data is a result of a deterministic imputation, which ignores the unexplained variation across households. Reproducing the distribution with a regression model requires adding an error term that captures the unexplained variation in the data. The first implementation of the indirect tax tool in EUROMOD is based on a linear model that relates the log of consumption expenditure to household characteristics (De Agostini et al. 2017). An interesting alternative is suggested by López-Laborda et al. (2020), who argue that the re-transformation of log consumption to nominal consumption expenditure, in the presence of heteroscedasticity, results in biased estimates. They suggest the use of generalized linear models and show that a GLM model with the log as link function and a distribution from the gamma family delivers the best results.

Matching methods have several advantages, among them that the imputed values inherit the distribution including the unexplained variation from the original data. For experimental data on income, consumption and wealth, Lamarche (2017) use a hot deck matching procedure, which uses for each household in the income survey the consumption expenditure of a comparable household in the consumption survey. The newest version of the indirect tax tool in EUROMOD uses a combination of model based approaches and an hot deck matching approach (Akoğuz et al. 2020). First, a regression method is used to impute consumption in EU-SILC and the data from consumer expenditure surveys (the source data for consumption). Second, the imputed values are used to match households in EU-SILC with those in the consumer surveys. The final imputation is not the predicted values from the first step, but the actual values in the matched households. The method is implemented in EUROMOD, but unfortunately not for Austria.

The literature provides no clear indication that one method would outperform others. We therefore applied several of these imputation methods and evaluate them according to how well the imputations reproduce the estimates of age-specific consumption from the original CES data. We use a linear model for the log of consumption, a GLM model with the log as link function and gamma distribution as well as hot deck matching of households. For our purpose a simple hot deck matching seems more appropriate as we aim to match households with similar characteristics, which is not guaranteed with the two-stage hot deck method. For all the methods we applied we use the same variables as explanatory characteristics of the level of consumption: household income per household member, the number of household members, the number of children below six, the number of children aged 6-14, and the number of persons of age 70 and older. Most research uses detailed characteristics of households, such as region and education level. However, these variables do not add to the explanatory power of the model once income and household size is accounted for and they do not change the results of our analysis. We therefore decided for a more parsimonious model. Details for each imputation method as well as the results of the regression models are given in Appendix 5.1.

The results of our comparison of imputation methods indicate only minor differences between the various approaches (Fig. 8 in the Appendix). We finally decided for the matching method because it is the most intuitive one and the associated age-profile had the smallest absolute deviation from the age-profile based on original data.

### Private market transfers: methodology for Austria 2015

After imputing consumption at the household level, consumption of households is allocated to its members based on the NTA consumption equivalence scale. This equivalence scale assumes that children until age four represent 0.4 equivalent consumers and for persons between age 4 and age 20 the equivalence scale increases linearly to one. After allocating household consumption to individual members, individual income and consumption is adjusted so that age and gender-specific values correspond exactly to the NTA age-profiles. This ensures that the estimates of intra-household transfers are consistent with the system of NTAs, i.e. that income plus public net transfers plus intra-family net transfers equals exactly consumption and saving at each age.

We need to emphasize that the estimates of consumption of individual household members and consequently the size and direction of intra-family transfers is influenced by the NTA consumption equivalence scale. The use of equivalence scales however is a common approach for evaluating costs of children (Humer and Rupp 2020) and the NTA scale has been intensively discussed within the NTA network. It represents a consensus that is based on the available evidence from different countries and facilitates cross-country comparisons, because the results are not influenced by country-specific methods and data for estimating consumption of children. Its basic features such as the strong increase of consumption with the age is also confirmed by the newest study of the costs of children in Austria (Bauer et al. 2021).

The further algorithm for estimating intra-household transfers in Austrian NTAs deviates slightly from the methods suggested in the NTA manual, because of the adjustments required for a gender-specific estimation and particularities of the data. We assume that income is shared within couples. The sharing among couples constitutes the first component of the intra-household transfers, and represents mostly transfers from fathers to mothers, because the latter reduce their employment (and income) because of care responsibilities. In the next step we calculated the difference between consumption and the shared income. If consumption exceeds income, it is assumed that the difference is financed by household members with income exceeding consumption. We assume that consumption of children is always financed by intra-family transfers, even when total income of households falls short of consumption and there is no household member with excess income. This assumption is necessary, because information on consumption expenditure is only collected for a two-week period and therefore subject to high random variation; the extrapolated consumption expenditure to yearly values frequently exceeds yearly income of households. Likewise, in other households the extrapolated consumption underestimates yearly expenditure. With our approach of accounting for transfers even when consumption estimates exceed income we receive unbiased estimate for transfer averages by age and parental status. This component of intra-household transfers constitutes mainly a redistribution from parents to children.

The main difference between the algorithm suggested in the NTA manual and Austrian NTA is the lack of an explicitly defined household head. The algorithm suggested in the UN, (2013) is quite complex and gives the household head a central role. It is assumed that only the household head owns asset. This assumption affects intra-household transfers, because all income that is not used for consumption needs to be transferred to the household head for saving, and only the household head can finance the consumption of children if total household income falls short of consumption. We decided to adapt the algorithm because it is likely to bias the gender-specific results. We know from a special module in the Household Finance and Consumption Survey that most assets are equally distributed among couples rather than belonging to a single person in the household (Groiß et al. 2018). By assigning all assets to a single person in the household and making her to the central person regarding intra-family transfers, results of gender-specific NTAs are likely to be strongly influenced by the choice of the household head. Because of this concern we treat all adult members in the same way. Our approach aims to improve the gender-specific estimates, but does not limit the comparison of non-gender-specific NTAs with the data from other countries.

A further difference to the guidelines in the NTA manual is that inter-household transfers are not estimated in Austrian NTAs for 2015. Data on inter-household transfers between generations in Austria are of low quality and do not permit age-specific estimates. As we mentioned above, the transfers measured in NTAs include only ”current transfers”. Wealth transfers, such as bequests, are not included. The negative residual (”saving”) for young adults (Table 1 and Hammer (2020a)) suggests that current transfers and wealth transfers between households may play an important role in financing consumption in young adulthood. Negative values indicate that part of consumption is not financed out of income and most likely through transfers, because Austrians usually do not finance their consumption through credit. Unfortunately, the limited data availability prevented a more detailed analysis of inter-household current transfers and of wealth transfers.

### Private non-market transfers: national time transfer accounts methodology

National Time Transfer Accounts measure production, consumption and transfers of services produced by non-market work. The most important non-market services include cooking, cleaning, shopping and childcare. Non-market production of households is not captured in the National Accounts core system, but is occasionally estimated in so called household satellite accounts (Poissonnier and Roy 2017; Communities 2003). The estimations are challenging, since there are no data on the output of non-market production activities of households, let alone their market value. Furthermore, there is not even data on the value of the inputs, consisting mostly of unpaid work. Most household satellite accounts measure non-market production by valuing the time input with wage rates for comparable activities.

Non-market production for other household members is not only an important part of total production, it constitutes a fundamental type of transfer between gender and generations. Donehower (2019) developed the NTTA methodology to integrate non-market work into the NTA framework. NTTAs measure production, consumption and transfers of non-market services by age and by gender. We estimate NTTAs for Austria using the most recent time use survey (TUS) of 2008/09^4^.

While allocating non-market production to individuals is straightforward, the estimates of non-market consumption and non-market transfers require several assumptions. TUS micro-data provides information on time use for non-market production at individual level. Production estimates by age, gender or parental status simply use this information. However, the data do not provide information about who in the household consumes the produced services. To allocate non-market consumption to individuals, NTTAs use several methods and assumptions. These methods depend on the type of the activity and whether it is carried out for household members or other households. Non-market transfers are calculated as difference between production and consumption.

NTTAs assume that each household member profits equally from general household services such as cooking or cleaning. Unfortunately, we cannot observe who in the household consumes a certain service, the assumption that all members profit equally represents a reasonable minimal consensus that is applied in all countries and thereby ensures the comparability of NTTA data across countries. Consequently, consumption of household services that are produced for the own household is estimated by adding up the time used for production within households and distributing it to all members in equal shares. The consumption of non-market services produced for other households assumes an equal distribution over the total population, i.e. independent of age and gender. Non-market services for other households include voluntary work for non-profit organizations as well as help in household work.

Most childcare services are consumed by children in their very first years of life. To estimate the consumption of childcare services provided by household members, we assign the total time used for childcare in the household to the children. This approach is straightforward if there is only one child in the household. In case there are more children in the household, we use age-specific weights. The age-specific weights account for the higher need of younger children and are based on age-specific amount of childcare in households with only one child. The Austrian TUS includes information on childcare provided for other households. We allocate these care activities to the children outside their own household using age-specific weights, which are derived from information on the use of childcare in EU-SILC. The weights reflect that non-household members, such as grandparents, use more time to take care of older children and less for very young children. For adult care we assume that it is provided to the oldest person in the household. In general, total time that is identified as adult care in the time use survey is small.

To assign a monetary value to non-market work we value each hour of non-market work either with the minimum wage rate for housekeepers or childcare workers. The activities other than childcare are valued with the hourly costs of a housekeeper employed at minimum wage in 2015, accounting for Christmas and holiday allowances, holidays and the employers social contributions. Childcare is valued with the costs of a childcare worker, which is somewhat lower than the costs for a general housekeeper.^5^ Donehower (2019) suggests the use of average wages to value unpaid work. Because we assume the minimum wage of experienced workers and top-ups for household staff over minimum wages are low, these values should represent a good approximation for average wages in this sector. The rationale behind the use of gross wages for valuing non-market work is that a shift from non-market work to market work should not affect the measure of total production and transfers. If parents decide to employ a nanny instead of providing childcare by themselves, this should not change the transfer measure. Since parents must pay gross wages for the nanny, we must value their own work by the same amount.

## Results

The results for private market and private non-market transfers are presented separately and then combined into overall private transfers. Within each step we first present age and gender-specific per capita transfers. Second, we present the total volume of age and gender-specific transfers in Austria. And third, we analyse transfers by parental status. The terminology *net transfer benefits* is used for benefits less contributions and *net transfer contributions* for the contributions less benefits.

### Private market transfers

**Fig. 1:**
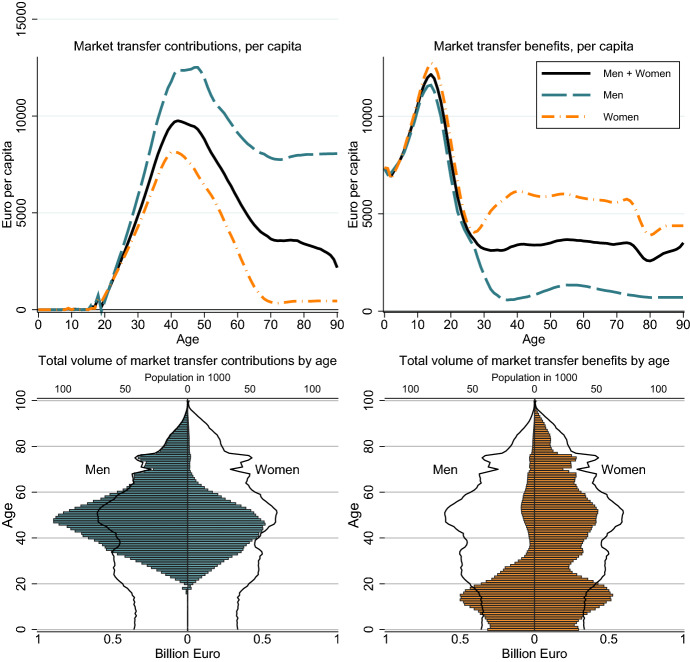
Private market transfers by age and gender in Euro. Source: Authors’ own calculations

Intra-family market transfers consist mainly of transfers from parents to children and transfers between partners. Market transfer benefits peak around age 15 with mean values of more than 11,000 Euros (Fig. 1, upper right panel). At this age young persons require high consumption expenditure but are still dependent on their parents. With the entry into the labour force and own income, market transfer benefits decline strongly. However, on average, adult women continue receiving market transfer benefits of more than 5000 Euro per year. This pattern reflects the specialization of fathers in paid work and of mothers in household work and childcare. Furthermore, the lower income of women translates into lower pensions and continued private transfers from men to women during retirement. The market transfer contributions peak at age 35–50 with values of more than 10,000 Euros for men and 5,000 for women (Fig. 1, upper left panel).

To illustrate the macro-economic importance of intra-family transfers we calculate total market transfer contributions and benefits by age groups and gender (Fig. 1, lower panel). Total market transfers within households amount to 41 billion Euros and correspond to 17 per cent of primary income. About 47 per cent of the market transfers are provided to the population 0-24, corresponding to 19 billion Euros. About 43 per cent of the market transfers are received by adult women (aged 25+) and 9 per cent by adult men. Because of their higher income, men provide 71 per cent of total intra-family market transfers, women 29 per cent.

### Private market transfers by parental status

**Fig. 2:**
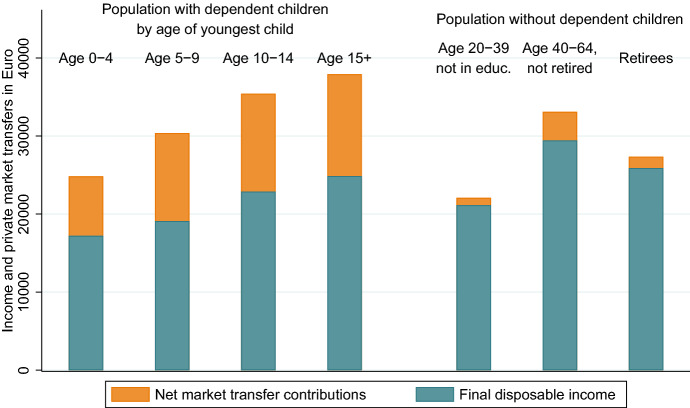
Decomposition of disposable income into net market transfers and final disposable income. The height of the bar represents individual disposable income before intra-family transfers. Source: Authors’ own calculations

The value of private market transfer contributions is strongly related to parental status and age of the youngest child. The relation between age and private transfers reflects the age-specific share of parents with dependent children in the population. Within age groups there are large differences in private transfer contributions, dependent on parental status. The information on private transfer contributions and benefits allow us to analyse the economic situation of parents accounting for support of children and their partner. We use the term *final disposable income (FDI)* for disposable income after accounting also for private market transfers (see Sect. 5.2 in the Appendix for an overview of the different income measures). It represents the amount of income available for own use, after fulfilling financial responsibilities for children and the partner. To illustrate the amount of income that parents use for children, we decompose disposable income (before paying/receiving private transfers) into the net contributions to private market transfers and FDI (Fig. 2). Because disposable income and transfers depend strongly on the age of parents and children, we distinguish parents by age of the youngest dependent child, and the population without dependent children by age and economic status. Dependent children are identified as those persons below the age of 30 who report education as their economic status. The difference between disposable income and FDI is much higher for parents with dependent children and represents their high net contributions to private market transfers.

Parents use about one third of their income for their children. Parents with young children have comparably low net hourly wages and additionally they have to restrict their labour force participation because of care responsibilities. Their average FDI is with less than 20,000 Euro considerably lower than for the other groups considered. Disposable income and FDI increases with age of the child and age of the parents. This is an effect of income generally increasing with age and of mothers reentering the labour market. However, the FDI of parents with dependent children above the age of 15 is with 24,000 Euros still considerably lower than for older working age adults and retirees without dependent children. Their FDI is about 29,000 Euros and 26,000 Euros, respectively.

### Private non-market transfers

**Fig. 3:**
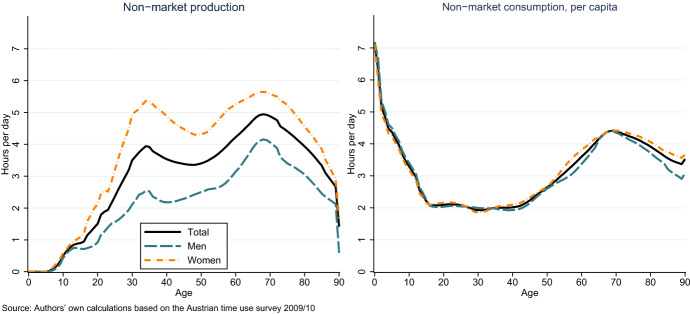
Non-market production and consumption by age and gender. Source: Authors’ own calculations based on the Austrian time use survey 2008/09

The production of non-market services is characterized by large differences between age-groups and gender. During working age, women use between 4 and 6 hours per day for non-market work, men slightly more than 2 hours (Fig. 3). For both men and women non-market work peaks around age 70 with about four hours for men and 5,5 hours for women. If this time is valued with wages of household staff and professional childcare, the value of non-market production and consumption amounts to 140 billion Euros, 58 per cent of primary income, or 40 per cent of GDP. These values are in line with estimates from other countries, which range between 30 and 60 per cent of GDP, depending on the specific method (Poissonnier and Roy 2017). In contrast to production, consumption of non-market services peak in the first years of life, corresponding to more than 6 hours of work per day. The working age population consumes services that require about two hours of non-market work per day. Consumption increases again towards retirement, in line with the higher production. The difference between consumption and production represents the net non-market transfers.

**Fig. 4:**
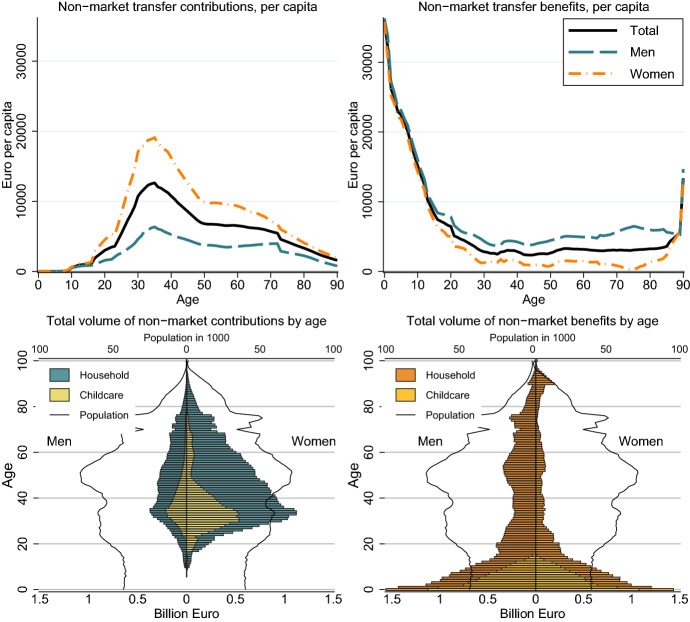
Private non-market transfers per capita by age and gender in Euro. Authors’ own calculations

Most of intra-family non-market transfers are provided by working-age women for their children and their partner. By contrast, elderly persons use non-market work to produce for themselves. The value of non-market services that are provided to children and the partner amount to more than 15,000 Euros per year for women at age 30-40 (Fig. 4, left panel). By contrast, the contributions of men are around 5,000 Euros at age 30-40, only slightly above the value they receive (Fig. 4, right panel). Children are the main receiver of non-market transfers, exceeding the value of 30,000 in the first years of live. Because monetary values are strongly dependent of the exact method of valuation, we provide the value of net transfers in terms of time in Fig. 10 in the Appendix. As a rule of thumb, about 5,000 Euros per year correspond to one hour of daily work. Young children receive services that require more than six hours of non-market work. These services are mostly provided by women at age 30-40, who use more than three hours of work per day for producing services for other household members.

Based on our valuation, total transfers in form of unpaid work correspond to 51 billion Euros or 21 per cent of primary income. The population below the age of 25 receive 60 per cent of the non-market transfers with a value of 31 billion Euros. Another group that receives large non-market transfers are adult men aged 20+: they receive 30 per cent of the non-market benefits, compared to adult women who receive only 10 per cent. In total about 74 per cent of the non-market transfers are provided by women.

### Private non-market transfers by parental status

**Fig. 5:**
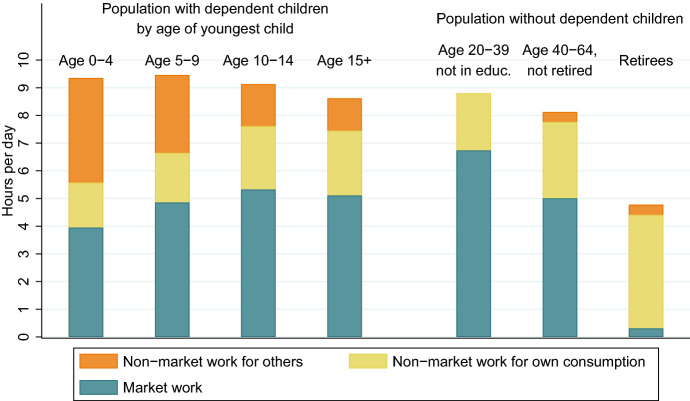
Total work by type and age of child. Source: Authors’ own calculations based on the Austrian time use survey 2008/09

For analyzing non-market and total work by parental status, total time used for work is decomposed into its components. We distinguish between paid work, non-market work for own consumption, and non-market work for others. The non-market work carried out for others represents the net non-market transfer contributions. These transfer contributions depend largely on the number and age of the youngest child in the household. Parents with children below the age of 4 provide four hours of non-market work to other generations (Fig. 5). To accommodate these responsibilities, parents reduce paid work and non-market services produced for themselves. Nevertheless, parents with young children are the group with the highest amount of time used for work in total. On average they use more than nine hours per day for work. Total working time and non-market transfers decreases with the age of the child, while market work and non-market work for own consumption increases. Retirees have the lowest level of total work. They do carry out a considerable amount of non-market work. In contrast to parents with small children, they produce these services mainly for their own consumption.

### Total private transfers

**Fig. 6:**
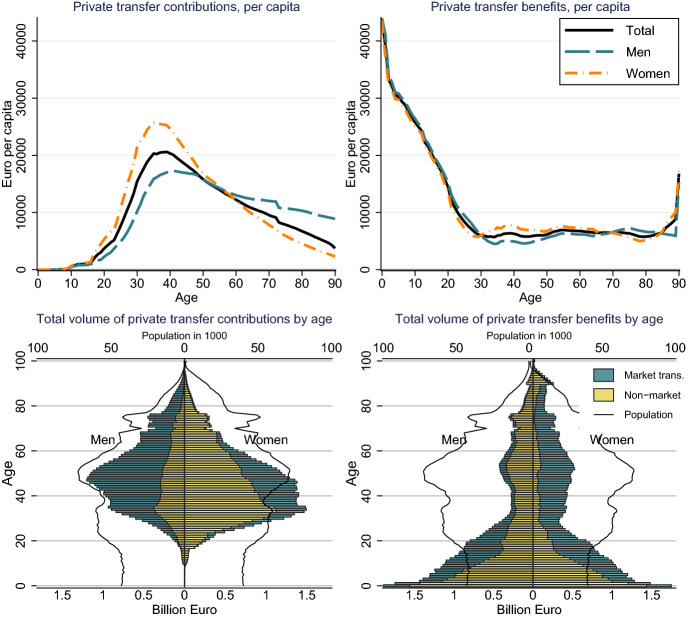
Total private transfers (market + non-market) by age and sex. Source: Authors’ own calculations

Combining the market and non market transfers allows a comprehensive illustration of total transfers within the family (Fig. 6). Most of the needs of children are covered by private transfers. Market and non-market transfer benefits together amount to 40,000 Euros in the first year of life and decrease to about 10,000 Euros at age 20. The specialization of women in non-market work and men in market work results in considerable transfers between partners. During working age men and women receive transfers from their partner with a value of about 8,000 Euros. The largest contributions are made at those ages when people have young children. Women provide most of the non-market transfers to young children. Total private transfers peak at age 30-40 with a value of 25,000 Euros. Men provide a larger part of the market transfers. More of these transfers are directed to older children and peak therefore at higher ages of the parents. For men the private transfer contributions peak at age 35-50 with about 17,000 Euros.

In total, the value of intra-family transfers amounts to 93 billion Euros. Of which 50 billion are provided to the population below the age of 25. To illustrate the size of this transfers a comparison with public transfers is helpful: net public transfers to children amount to 25 billion Euros, the net public transfers to the elderly population aged 60+ to 58 billion Euros. Total private transfers are rather equally distributed among gender, with women providing 51 per cent of total. Although women contribute more to private transfers until about age 50, men provide more private transfers in old age. This pattern illustrates the economic challenges for mothers in case of a break-up of their relationship: they provide the transfers to children, but loose the compensation from their partner for their lower market income and the smaller pension benefits.

### Total private transfers by parental status

**Fig. 7:**
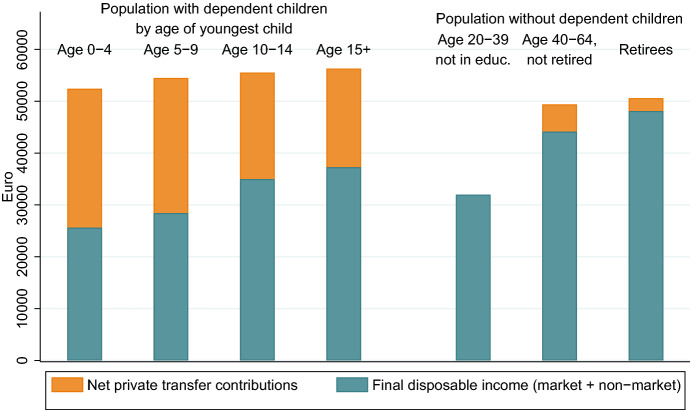
Disposable income and final disposable income: market and non-market. The height of the bars represent income and the value of non-market production. Source: Authors’ own calculations

**Fig. 8:**
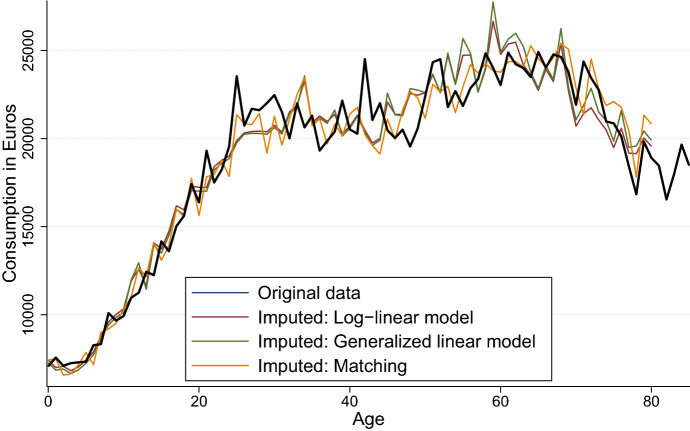
Comparison of imputation methods based on the age-profile of consumption. Sources: EU-SILC and consumer expenditure survey 2014/15 of Statistik Austria

**Fig. 10:**
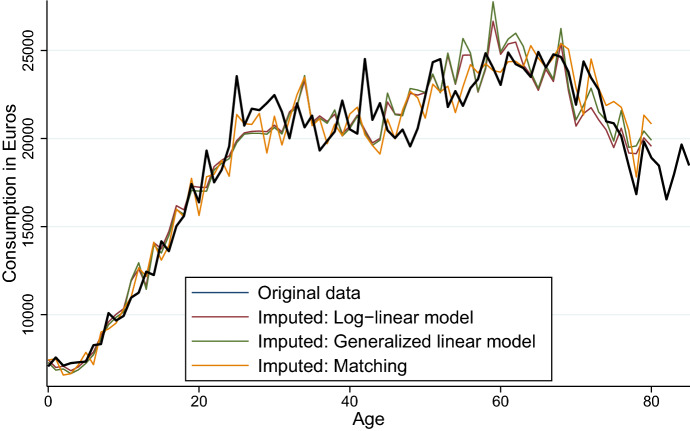
Net non-market transfers by age and gender in hours per day

Parents produce more than people without dependent children, they provide most of private transfers and end up with lower resources for themselves. Figure 7 shows net transfer contributions and final disposable income including non-market production by parental status. These two components together represent the total disposable income of parents before private transfers through unpaid work. Because of their higher non-market production, total income and production are higher for the group of parents. However, a large part of income is redistributed to their children. The total final disposable income is considerably lower for the group of parents.

## Conclusion

Transfers within families are a central component of the intergenerational transfer system. Based on NTA data for Austria 2015, we estimate that the value of total private transfers amounts to 93 billion Euros, corresponding to 38 per cent of primary income. Out of which the transfers to the population below age 25 amount to 50 billion Euros. For comparison, the net public transfers to the population 60+ had a value of 58 billion Euros in 2015. The estimates of private transfers include market transfers and non-market transfers such as care and household services. The value of market transfers to the population aged 0-24 amounts to 19 billion Euros, the value of non-market transfers is in the order of 31 billion Euros.

Intra-family transfers constitute a considerable economic burden for the parents. We refer to disposable income after intra-family transfers as final disposable income. Parents with dependent children use about one third of their income for their children by financing their consumption or by providing monetary transfers. These responsibilities for children results in a considerably lower final disposable income as compared to groups without dependent children. Parents with children below the age of five have a final disposable income of about 17,000 Euros, while final disposable income of retirees without dependent children is about 26,000 Euros and of older working age adults without dependent children more than 29,000 Euros per year. Furthermore, the care responsibilities for children result in a higher total amount of work. Parents with children below the age of five use about nine hours per day for market and non-market work. About four hours of this time are used to produce non-market services for the children.

Remarkable are the strong gender patterns in private transfers. Due to their higher income, men provide 71 per cent of the private market transfers. By contrast, women provide 74 per cent of the non-market transfers. Men share their higher income with their partner even after children have left the joint household, while women continue providing non-market transfers in form of household work. Because of their lower income and lower contributions to the social security system, women end up with lower pensions than men. This pattern explains the high risk of poverty for lone mothers and women in old age. With a break-up of their relationship they loose access to private transfers as important component of their disposable income.

Understanding economic vulnerability and inequality requires the consideration of private transfers. When discussing the social system and reform options of the welfare state in the context of demographic change, we often solely focus on public transfers, ignoring the role of private transfers. For societies private transfers constitute important human capital investments. The costs are paid almost exclusively by parents. For parents the transfers to children are synonymous to additional expenditure, lower labor force participation, lower income and they consequently result in lower pension rights in old age. Not surprisingly, families are among the groups with the highest risk of poverty. In couples the economic burden of children is shared and mitigated by specialization and transfers between partners. A break-up of the relationship is associated with a loss of access to private transfers, resulting in low income and high poverty among lone parents and women in old age. For a complete picture of the economic situation of different generations and different groups in our society it is of utmost importance to consider the important role of private transfers.

## Figures and Tables

**Fig. 9:**
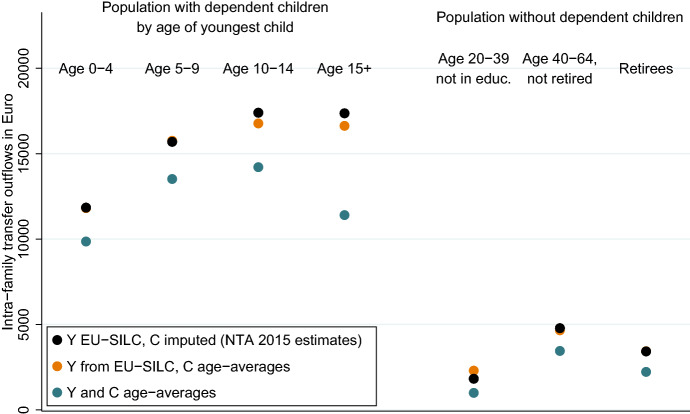
Intra-family market transfer contributions: comparison of imputation of age-averages and imputation using hot deck matching for consumption (C) and income (Y)

## Data Availability

The age and gender specific data and a detailed description are available at https://doi.org/10.11587/4EOXZO.

## Appendix

### Imputing consumption into EU-SILC 2015 for Austria

The NTA methodology for estimating intra-family transfers requires microdata with information on income at individual level and consumption at household level. This requires the combination of two data sources: EU-SILC includes information on income of individuals, but no information on consumption. The consumer expenditure survey, in turn, includes information on income and consumption, but neither one at the individual level. We use a log-linear model, a GLM model with log-link and gamma distribution, and a hot deck matching approach to impute information on consumption into EU-SILC. The methods are evaluated based on how well the age-profiles of imputed data fits to the estimate of the age profile in the original data.

#### Regression models

Modelling log consumption as linear function of income and other household characteristics is the most common approach (e.g. De Agostini et al. 2017). López-Laborda et al. (2020) suggest the use of GLM models because they can better deal with heteroscedasticity. In both models we use income, income squared, household size (number of members), the number of household members below age 6, the number of household members aged 6-14, and the number of household members of age 70 and older. Income and household size are by far the most important determinants of consumption. Other variables such as age-structure of households, education level, or location are significant, but after controlling for income they add little to the explanatory power of the model. We decided however to use the information on the age-structure of households in addition to income and the size of households, because it slightly improves the imputation for our purpose of analysing age-groups and household types. Other variables are left out because they do not influence our results (Table 2).

**Table 2:** Regression models for imputing consumption in income data

	(GLM)	(Log-linear model)
	Consumption per capita	Log consumption per capita
Household inc. per capita	0.0000322***	0.0000326***
	(34.86)	(879.22)
HH inc. per capita squared	-8.36e-11***	-9.84e-11***
	(-15.24)	(-333.35)
No. household members	-0.0997***	-0.0895***
	(-13.77)	(-324.33)
No. children aged 6-14	-0.0270	-0.0298***
	(-1.66)	(-48.60)
No. children below age 6	-0.0675***	-0.0650***
	(-3.81)	(-97.12)
No. members aged 70+	-0.106***	-0.115***
	(-8.77)	(-249.70)
_cons	9.594***	9.457***
	(364.24)	(9375.30)
$N$	6861	6861

*t* statistics in parentheses

*$$p<0.05$$, **$$p<0.01$$, ***$$p<0.001$$

The log-linear model and the GLM model deliver similar results with log consumption increasing almost linearily with income. With comparable income per capita, larger household consume less, indicating economies of scale regarding consumption expenditure. Household members of child age or of old age are associated with lower household consumption compared to adult members of age 15–69.

#### Matching methods

The similarity measure between households accounts for the income percentile, the number of household members, the number of children below age of six, the number of children aged 6-14 and the number of household members aged 70 and older. To match households from the CES to households in EU-SILC we use the Stata tool *teffects*, which identifies for each household in the income dataset the nearest neighbours in terms of the Mahalanobis distance. To avoid that the same household from the CES is matched to several households in EU-SILC, we use an algorithm that selects the nearest possible neighbour under the condition that each household in the consumer expenditure survey is matched at maximum twice. It would be possible to allow only one match, because CES includes more households than EU-SILC. However, such algorithm that allows only one match results in considerably difference between some of the matched households, when the nearest neighbours are already selected for other households.

#### Evaluation

We evaluate the different imputation methods regarding the similarity of age profiles based on the imputed data with the age profile based on the original data (Fig. 9) The differences are extremely small and hardly visible in the plot. The total absolute deviation (sum over all age-groups) is smallest for the matching method, but the performance depends more on the exact specification of the regression model and the matching algorithm than on the imputation method. In particular, the log-linear model delivers robust and good results and the GLM model never outperformed the log-linear model.

#### Comparison: combining income and consumption data with age-averages and with proper imputation

Our imputation approach does improve the intra-family transfer estimates, compared to the use of age-specific means. In Fig. 9 we plot the estimates of intra-family transfer outflows by household type, comparing our imputation with the use of age means as suggested in the NTA manual. It is clear that imputation of income and consumption with age and gender-specific means (Y and C age-averages) does not allow a separate estimation of intra-family inflows and outflows. The estimates are biased towards zero, as highlighted in UN, (2013). If only consumption is imputed using age means the bias is small. The bias is highest (but still rather small) in the group with dependent children above 15. Older, dependent children living with their parents are likely to have lower consumption than their peers which are already living in their own households, also reflecting the economies of scale in consumption. Age-specific means are likely to overestimate their consumption level and result in lower estimates of intra-family transfer contributions for the parents. However, the differences are small and indicate that the algorithm is robust with regard to the imputation method of consumption in income data.

### National transfer accounts for Austria 2015, extended table

Table 3 provides an overview of the sequence of NTAs (similar to the sequence of accounts in NAs), as described in Sect. 3.1. Starting from primary income (the equivalent to national income in the SNA) individuals (arranged by age-groups) pay contributions to the public transfer system and receive benefits. The income after public redistribution is referred to as disposable income. Out of disposable income individuals contribute to the intra-family transfer system or receive benefits out of it. For income after accounting for private redistribution we use the term *final disposable icnome*. It has no equivalent in SNA or income surveys, as these data do not account for private redistribution. To fully account for public redistribution we have to add public consumption to our income measure, which we then call adjusted disposable income. This terminology is inspired by the terminology used in SNA, but adjusted disposable income in the NTA context includes all public consumption, while adjusted disposable income usually only accounts for social benefits in kind (individual public consumption).

**Table 3:** National transfer accounts for Austria 2015: Yearly values in Euro, three age groups. Extended table

	Age 0–24	Age 20–59	Age 60+
**Primary income (labour + asset)**	**5289**	**45,449**	**17,185**
Public transfer contributions	4395	23116	9935
Public transfer benefits in cash	610	3853	18661
**Disposable income**	**1505**	**26,185**	**25,911**
Private transfer contributions	389	7456	3916
Private transfer benefits	8690	3471	3343
**Final disposable income (FDI)**	**9806**	**22,200**	**25,338**
Public transfer benefits in kind	10,100	5517	8956
**Adjusted FDI**	**19,906**	**27,717**	**34,294**
Private consumption	10,265	18,554	20,484
Public consumption	10100	5517	8956
Residual (saving)	− 459	3645	4853
Population	22,36,257	43,21,608	21,26,450

### Transfers in hours per day

See Table 4

**Table 4:** Data for Figs. 2, 5 and 7

	Population with dep. children by age of youngest child				Population without dependent children		
	Age 0–4	Age 5–9	Age 10–14	Age 15+	Age	Age	
					20–39	40–64	
					Own inc.	Not retired	Retirees
**Figure 2: Final disposable income and private market transfers (Euro per year)**							
Disposable income	24,833	30,371	35,424	37,913	22,072	33,098	27,346
Private market transfer contributions, net	7,624	11,266	12,546	13,045		3,665	1,466
Final disposable income	17,209	19,105	22,878	24,868	21,140	29,434	25,881
**Figure 5: Total work and private non-market transfers (hours per day)**							
Total work	9.4	9.5	9.1	8.6	8.8	8.1	4.8
Non-market work for others	3.8	2.8	1.5	1.2		0.4	0.4
Non-market work for own consumption	1.6	1.8	2.3	2.3	2.1	2.8	4.1
Market work	3.9	4.9	5.3	5.1	6.7	5.0	0.3
**Figure 7: Total FDI and total transfers (market + non-market, Euro per year)**							
Total disposable income (m + nm)	52,401	54,483	55,531	56,300	32,917	49,377	50,580
Net transfers to others	26,785	26,073	20,566	19,048		5,243	2,500
Final disposable income (m + nm)	25,617	28,410	34,965	37,252	31,985	44,134	48,080

.

## Footnotes

1 A transfer is defined as a transaction in which one person provides a good, service or asset to another person, without receiving from the latter any good, service or asset in return as counterpart (cf. SNA 2009). It includes in particular public pensions and parents financing the consumption of their children. Assets are accumulated to generate income in the future, to be sold or bequeathed later in life. Asset based reallocation constitutes therefore an important instrument to reallocate resources across life stages.

2 The Austrian NTA data for 1995, 2000, 2005 and 2010 can be accessed at www.ntaccounts.org, the European NTA and NTTA data at www.wittgensteincentre.org/ntadata.

3 Konsumerhebung 2014/15 of Statistik Austria.

4 Statistik Austria: Zeitverwendungserhebung 2008/09.

5 The gross monthly minimum wage in 2015 was 1,436 Euro for a housekeeper without professional education and 6 years of experience (http://www.ris.bka.gv.at/eli/bgbl/II/2014/299). With the non-wage labour costs, consisting mainly of social contributions paid by the employer (http://rechner.cpulohn.at/bmf.gv.at/), and a 1,722 yearly working hours (http://wko.at/statistik/eu/europa-arbeitszeit.pdf) the hourly wage is 15.28 Euro. With a similar calculation we optain a value of 13.33 Euro for one hour of childcare.

## Declarations

### Code availability

The code is available upon request.
